# Tricuspid valve endocarditis in a horse owner, caused by *Streptococcus equi* subsp. *zooepidemicus*

**DOI:** 10.1128/asmcr.00059-25

**Published:** 2025-07-31

**Authors:** C. Nicholas Roy, Catherine E. Wiechmann, Ameesh Dev, Brandon K. Walther, James M. Musser, Randall J. Olsen, Stephen B. Beres, Dierdre B. Axell-House

**Affiliations:** 1 Department of Medicine, Houston Methodist Hospital 828032, Houston, Texas, USA; 2 Department of Biomedical Engineering, Texas A&M University 549134, College Station, Texas, USA; 3 Department of Pathology and Genomic Medicine, Houston Methodist Hospital 735417 , Houston, Texas, USA; 4 Center for Infectious Diseases, Houston Methodist Research Institute 167626 , Houston, Texas, USA; 5 Department of Pathology and Laboratory Medicine, Weill Cornell Medical College and Cornell-Presbyterian Medical Center 12295 , New York, New York, USA; 6 Division of Infectious Diseases, Department of Medicine, Houston Methodist Hospital 23534 , Houston, Texas, USA; 7 Department of Medicine, Weill Cornell Medical College 12295 , New York, New York, USA; Vanderbilt University Medical Center, Nashville, Tennessee, USA

**Keywords:** group C streptococci, zoonosis, infective endocarditis

## Abstract

**Background:**

*Streptococcus equi* subsp. *zooepidemicus* (SESZ) are zoonotic group C streptococci primarily acquired from contact with horses and other animals, such as llamas. They are unusual causes of infection in humans and rarely cause infective endocarditis.

**Case Summary:**

A 58-year-old woman presented with fever, malaise, and polyarthritis. Clinical evaluation diagnosed native tricuspid valve SESZ endocarditis. The SESZ isolate was genetically closely related to a clone causing an outbreak of post-streptococcal glomerulonephritis linked to the consumption of unpasteurized cheese in Brazil. The patient had no exposure to unpasteurized cheese but rode horses. Her infection course was notable for persistent fever despite combination antibiotic therapy. Resolution of her symptoms ultimately required tricuspid valve and aortic valve replacement and mitral valve repair.

**Conclusion:**

This is the first reported case of native tricuspid valve SESZ endocarditis. The case demonstrates the natural history of this rare disease and illustrates the importance of taking an exposure history.

## INTRODUCTION

Streptococci once caused the majority of infective endocarditis (IE) cases. However, with the recent rise of *Staphylococcus aureus* endocarditis, streptococci now comprise 20%–30% of cases (1). The majority of streptococcal endocarditis cases are caused by viridans group streptococci, followed by Lancefield group D streptococci (*S. bovis/gallolyticus*) (2). Lancefield groups A, B, C, and G beta-hemolytic streptococci are uncommon causes of endocarditis (2). Group C and group G streptococci (GCGS) are closely related genetically, and the taxonomy of these organisms has been in flux over time (3), complicated by the lack of consistency in obtaining accurate species-level identification until recent advancements in matrix-assisted laser desorption/ionization-time of flight mass spectrometry (MALDI-TOF) (4). The GCGS *S. dysgalactiae* subsp. *equisimilis* and subsp. *dysgalactiae* are commensal organisms of the human oropharynx, skin, and female genitourinary tract, and they cause the majority of human GCGS infections (5). *Streptococcus equi* subspecies *zooepidemicus* (SESZ) and subspecies *equi* (SESE) are zoonotic GCGS; SESZ are commensal organisms of horses but can also cause acute equine infections (6). Human infections with SESZ can be acquired from contact with infected animals, primarily horses (6), and consumption of unpasteurized dairy products from animals with SESZ mastitis (7). We present a case of SESZ infective endocarditis in a patient with exposure to horses.

## CASE PRESENTATION

A 58-year-old woman presented with progressively worsening fever, malaise, and chills for 4 weeks and severe arthralgias for 4 days. She developed anterior right shoulder pain 4 days prior to presentation and left wrist pain and hand swelling 2 days prior to presentation. She had no additional symptoms, no history of surgeries or trauma, and no family history of autoimmune conditions. She was taking olmesartan for hypertension and sumatriptan for migraines but was otherwise healthy. Upon inquiry of potential exposure history, she stated that she had regular interaction with horses as an owner and also interacted with llamas and alpacas regularly at a farm. None of the horses or other farm animals were noticeably ill. On examination, she was tachycardic with a heart rate of 101 bpm and febrile with a temperature of 101.1°F. She had a grade II systolic murmur heard best at the left lower sternal border, decreased abduction of her right shoulder, and nonpitting edema of her left wrist and dorsum of her hand. Despite the fever, she did not appear acutely ill. The remainder of her physical examination was normal. Complete blood count was remarkable for white blood cell count of 13,000 cells/µL (ref. range, 3,400–11,000 cells/µL) and thrombocytopenia of 65,000 platelets/μL (ref. range, 150,000–450,000 platelets/μL). A complete metabolic panel showed serum albumin of 2.9 g/dL (ref. range, 3.5–5.0 g/dL), alanine transaminase of 63 U/L (ref. range, 0–50 U/L), aspartate transferase of 80 U/L (ref. range, 0–35 U/L), and was otherwise normal. Urine dipstick was positive for bilirubin, trace ketones, and 2+ protein. No bacteria were grown from urine. A chest radiograph was normal. Magnetic resonance imaging (MRI) of the patient’s right shoulder and left wrist demonstrated nonspecific inflammation of the acromioclavicular joint and synovitis at the base of the left thumb joint. Two sets of blood cultures were drawn, and cefepime 2 g q8h and vancomycin 15 mg/kg q12h after a one-time 20 mg/kg loading dose were initiated for empiric treatment of sepsis. All four blood culture bottles grew group C streptococci after 20 h of incubation. Species identification by matrix-assisted laser desorption/ionization-time of flight mass spectrometry (MALDI-TOF MS) in the clinical microbiology laboratory confirmed *Streptococcus equi* subspecies *zooepidemicus* (SESZ).

The genome of the SESZ strains recovered from the patient’s blood cultures was sequenced using an Illumina Nextera XT kit and NextSeq instrument as previously described (8). We retrieved 684 publicly available SESZ genomes, recovered from a variety of human and animal hosts worldwide, deposited in the NCBI Short Read Archive (https://www.ncbi.nlm.nih.gov/sra) and Microbial Genome Database (https://www.ncbi.nlm.nih.gov/genome/microbes). Phylogenetic analysis was performed to compare the patient’s SESZ isolates to the publicly available genomes.

**Fig 1:**
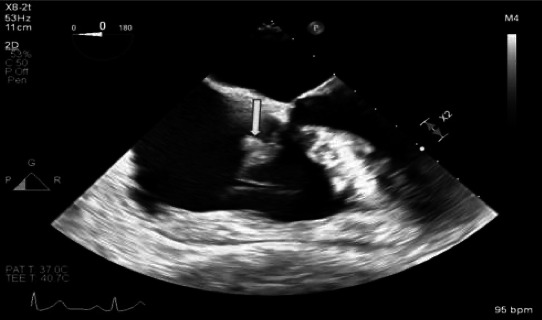
Tricuspid valve with 1–2 cm vegetation (yellow arrow) on the atrial surface.

Transesophageal echocardiogram (TEE) revealed several 1- to 2-cm vegetations on the atrial surface of the tricuspid valve with possible leaflet perforation (Fig. 1) and moderate tricuspid regurgitation. Antibiotic treatment was changed to ceftriaxone 2 g q24h and gentamicin 3 mg/kg q24h in accordance with the Infectious Diseases Society of America/American Heart Association guidelines for group C streptococcus endocarditis (9). Blood cultures were repeated 3 days after initial blood cultures were negative for growth. Cardiothoracic surgery consultants advised antibiotic treatment with follow-up at the end of therapy. Given the high suspicion for septic arthritis, joint aspiration of the left wrist triscaphe joint was performed, but the aspiration did not yield any fluid.

**Fig 2:**
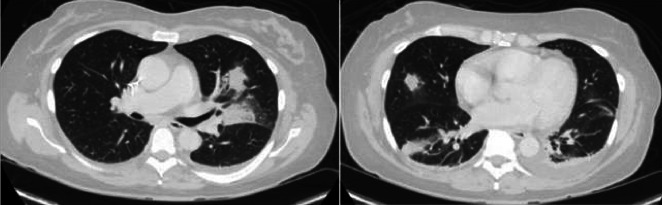
Computed tomography of the chest demonstrating multiple pulmonary opacities consistent with pulmonary septic emboli.

Within 3–4 days of antibiotic therapy, the patient had rapid improvement in her joint pain and swelling. Despite this, daily fevers up to 103°F persisted, and a computed tomography (CT) scan of chest, abdomen, and pelvis demonstrated mild multifocal pulmonary opacities consistent with septic emboli (Fig. 2). Gentamicin was stopped after the patient developed a mild acute kidney injury during her admission, which subsequently resolved. She continued to feel well, had resolution of leukocytosis, thrombocytopenia, and acute kidney injury, became afebrile, and was discharged on hospital day 12 on combination therapy with intravenous ceftriaxone 2 g q24h and oral levofloxacin 750 mg daily to complete 6 weeks of antibiotics.

**Fig 3:**
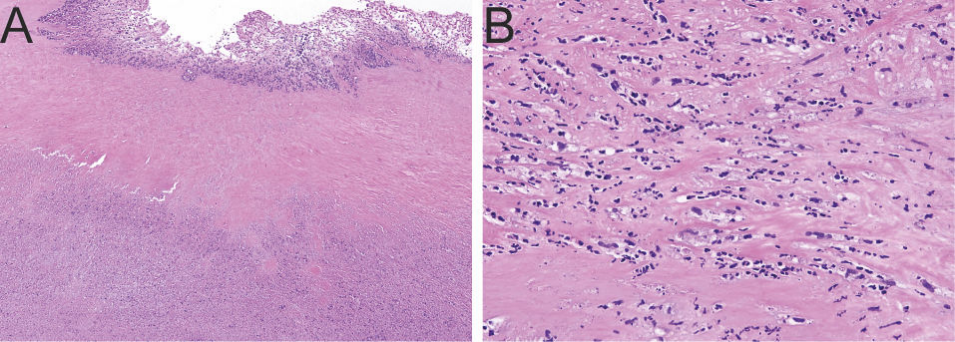
Histopathologic examination of the tricuspid valve leaflet. Micrographs show prominent areas of myxoid degeneration and necrosis with acute inflammation. Original magnification (**A**) 2× and (**B**) 20×. Hematoxylin and eosin (H&E) stain.

Upon follow-up in the Infectious Diseases clinic at the end of antibiotic treatment, the patient now had new symptoms of bilateral lower extremity swelling, shortness of breath, and subjective fever both during and after antibiotic treatment. Repeat TEE demonstrated failure of the lateral commissure of the tricuspid valve with associated worsened tricuspid regurgitation and new moderate mitral and aortic valve regurgitation. No abscess was apparent by TEE. The patient was hospitalized and found to have a left pleural effusion; 900 cc of exudative pleural fluid was removed with cultures yielding no growth. While hospitalized, she had recurrent daily fevers up to 102°F while remaining otherwise asymptomatic and without other abnormal vital signs. Blood cultures repeated on day 42 after the initial blood cultures remained negative. CT angiography of chest, abdomen, and pelvis, magnetic resonance imaging (MRI) of the brain with contrast, and a tagged white blood cell scan did not demonstrate evidence of disseminated infection. A cardiac MRI demonstrated new evidence of a flail mitral valve. Due to the patient’s worsening symptoms and valvular dysfunction, she underwent tricuspid valve and aortic valve replacement, mitral valve repair, and left atrial appendage clip. Tricuspid valve histopathology demonstrated marked necrosis with acute neutrophilic infiltrate (Fig. 3). She tolerated surgery well, was extubated on post-operative day 1, and was discharged home to complete a 4 week course of ceftriaxone 2 g q24h. Overall, she completed 10 weeks of antibiotics. She had complete resolution of symptoms of heart failure and fever and continues to do well more than a year after surgery.

## DISCUSSION

Human infections with SESZ can be acquired from consumption of unpasteurized dairy products from animals with SESZ mastitis (7), as well as contact with infected animals, primarily horses (6), and pertinently to our patient, alpacas and llamas (10), although direct animal-to-human transmission has not been documented. Our patient denied any obvious illness in animals that she encountered.

In the literature, human SESZ infections are predominantly reported in Europe, Asia, or Canada, with a variety of clinical presentations (6). Review of the modern epidemiology of SESZ native valve endocarditis identified eight cases published since 2000 (Table 1). Including our patient, the median age of patients with SESZ endocarditis is 65 years old. Two patients had exposure to unpasteurized cheese, four patients had exposure to horses, and one patient was a retired veterinarian. Four patients had an initial presentation with polyarthritis, with two patients with culture-proven SESZ septic arthritis. All prior reported cases had involvement of either the aortic or mitral valve (7, 11–17). To our knowledge, our patient is the first reported case of right-sided SESZ endocarditis. Our patient was previously healthy, with a past medical history of well-controlled hypertension and migraines. She had no previous cardiac history and was fit and active prior to illness. She did not have typical risk factors for right-sided endocarditis, including intravenous drug use, implantable cardiac device, or cardiac abnormalities.

**TABLE 1:** Characteristics and outcomes of published cases of patients with SESZ endocarditis since 2000^*a*^

Ref.	Age/sex	Country	Medical history	Presenting symptoms	Exposure	Valve involved	Treatment	Complications	Outcome
(11)	55/F	Romania	HTN, biliary dyskinesia	Fever, headache, confusion, obtundation	Unknown	Aortic	Penicillin G x 4 wk, gentamicin x 2 wk	Ischemic stroke	Survival
(9)	70/F	Spain	None	Fever, gastrointestinal symptoms	Unpasteurized cheese	nd^*b*^	Beta-lactam	Pneumonia	Survival
(12)	82/M	Denmark	T2DM, IHD, AFib, gout, prostate cancer	Dyspnea, hemoptysis, fever, severe left shoulder pain, LE edema	Horses	Aortic	Benzylpenicillin + gentamicin, followed by amoxicillin + rifampin, total 87 d	Left shoulder osteitis	Survival
(13)	79/M	South Africa	None	Left wrist and right knee pain/swelling, fever, headache, lethargy	Horse manure	Aortic	Benzylpenicillin x 6 wk	Left wrist and Right knee septic arthritis	Survival
(14)	65/M	United States	None	Fevers, bilateral ankle/knee pain and edema	Veterinary occupation	Mitral	Penicillin G followed by ceftriaxone, 8 wk	Polyarthritis, L5-S1 discitis/OM with epidural phlegmon	Survival
(15)	57/M	Finland	Bicuspid AV, AI	Fever, obtundation	Horses (horse breeder)	Aortic	IV penicillin x 5 wk, gentamicin x 10 d	Meningitis	Survival
(16)	59/M	Quebec, CA	HTN, T2DM, HLD, CAD, CKD, ASD	Generalized weakness, lightheadedness, dyspnea, cough, fever	Horses	Mitral	Ceftriaxone + rifampin, 6 wk	Bilateral endophthalmitis, blindness, meningitis	Survival
(17)	69/M	Colombia	None	Polyarthralgia, followed by right hemiparesis, obtundation	Unpasteurized milk	Mitral	Penicillin G	Hemiparesis	Deceased

^*a*^ AFib: atrial fibrillation, AI: aortic insufficiency, ASD: atrial septal defect, AV: aortic valve, CAD: coronary artery disease, CKD: chronic kidney disease, d: day(s), F: female, HLD: hyperlipidemia, HTN: hypertension, IHD: ischemic heart disease, IV: intravenous, LE: lower extremity, M: male, nd: not described, OM: osteomyelitis, T2DM: type 2 diabetes mellitus, wk: week(s).

^*b*^ nd, not described.

Of the publicly available SESZ genomes, the patient’s isolates were genetically most similar to strains recovered during a post-streptococcal glomerulonephritis outbreak epidemiologically linked to consumption of unpasteurized cheese (18). Our patient denied consumption of unpasteurized dairy products. Although her initial hospital course was complicated by mild acute kidney injury, this was temporally related to the initiation of gentamicin therapy, and renal abnormalities resolved after discontinuation of the antibiotic. She did not have any other episodes of impaired renal function or clinically apparent glomerulonephritis throughout her follow-up.

Our patient notably had persistent fever during her initial hospitalization and subsequent outpatient treatment and through her second hospitalization up until surgery. Persistent fever in patients with infective endocarditis (IE) is worrisome for unidentified disseminated sites of infection, cardiac complications such as cardiac abscess, new infections including nosocomial, and drug fever (19). However, none of these etiologies could be identified in our patient. We hypothesize that her persistent fever is related to the ongoing inflammation and necrosis of the large valvular vegetation, as described in other reports of IE (20). This possibility is particularly supported by her complete defervescence after surgery.

SESZ is closely related to group A *Streptococcus* (GAS, *S. pyogenes*) (18). While the molecular pathogenesis of GAS has been well studied, little is known about GCGS. A recent study of GCGS *S. dysgalactiae* subsp. *equisimilis* (SDSE) strains reported that compared to other SDSE emm-types, stG62647 strains were associated with significantly increased virulence in a mouse model of invasive infection (8). The molecular basis for the difference in virulence among SDSE strains is under investigation. The potential role of virulence factors shared among GAS and GCCS, such as secreted toxins, proteases, antiphagocytic proteins, and others, is unstudied in SESZ human disease.

In conclusion, *Streptococcus equi* subsp. *zooepidemicus* is a zoonotic pathogen with prominent risk factors of exposure to horses and other domestic animals and consumption of unpasteurized dairy products. Human infection with SESZ can be invasive and severe, and endocarditis is an uncommon but possible manifestation. Clinicians should have a high index of suspicion for zoonotic infections, such as those caused by SESZ in patients with significant exposure to animals. Further study is needed to understand the molecular pathogenesis of SESZ, which may reveal which patients are likely to have severe disease courses or suffer post-streptococcal immunological phenomena.

## Data Availability

Whole genome sequencing data for this investigation were submitted to the National Center for Biotechnology Information Sequence Read Archive under BioProject accession number PRJNA1134679.
